# Bunion

**Published:** 1898-02

**Authors:** 


### BUNION.

Parker Syms, of New York, makes an incision about an inch in length on the dorsum of the toe. In a mild case, after retracting the tendon of the extensor proprius pollicis outward, he chisels off all the overprominent portion of the inenr side of the head of the metatarsal bone, removing as much bone as is necessary to do away with all protuberance; he then sutures the wound and lets it heal under one dressing. Usually the patient can walk about after the first week.

In severe cases, where there is a marked adduction as well as lateral dislocation, Syms removes the head of the metatarsal with a chisel or bone-forceps, and also cuts off the prominent inner side of that bone. To resect the head of the metatarsal bone it will be necessary to divide the lateral ligaments and completely dislocate the toe. This can be done with ease and satisfaction through the simple straight incision described. It is necessary to remove so much bone that the toe will readily come into place and have no tendency to displacement. If this is not accomplished by the first ablation, more bone must be removed.

The dressing must be carefully done and close attention given to the aftertreatment, which should include the application of a plaster splint. The writer advises never to operate during an acute attack of inflammation; always to treat the deformity and never operate on the bursa, for it will take care of itself after its cause is removed (the exceptions to this rule are the removal of callosities from the bursa when they exist, and the incision of bursae when they suppurate); never to make the operation incision around or through the bursa.—*New York Medical Journal.*

